# Assessment of cervical cancer services and cervical cancer related knowledge of health service providers in public health facilities in Addis Ababa, Ethiopia

**DOI:** 10.1186/s13104-019-4701-6

**Published:** 2019-10-21

**Authors:** Firdawek Getahun, Adamu Addissie, Shiferaw Negash, Gebrekiros Gebremichael

**Affiliations:** 1 Department of Public Health, Arbaminch University, Arba Minch, Ethiopia; 2 School of Public Health, Addis Ababa University, Addis Ababa, Ethiopia; 3 Department of Obstetrics and Gynecology, Addis Ababa University, Addis Ababa, Ethiopia

**Keywords:** Cervical cancer services, Knowledge, Health service providers

## Abstract

**Objective:**

To assess cervical cancer services and knowledge of health service providers in public health facilities.

**Result:**

Two of the three hospitals had cervical cancer screening services. One-third of the hospital had cervical cancer diagnosis service punch biopsy and cervical cancer treatment. Majority, 289 (93.5%) of study participants said cervical cancer was a preventable disease. Having multiple sexual partners 257 (83.2%) and post coital bleeding 251 (81.2%), were the most mentioned risk factor and clinical manifestation of cervical cancer respectively. Majority of the participants were aware of the correct time to start screening 291 (70.5%), and only 95 (25.9%) knew the screening intervals. Overall, 165 (53.4%) of health providers scored below the mean knowledge level score. Females had better knowledge about cervical cancer than males (X^2^ = 8.4, P = 0.003).

## Introduction

Human Papilloma Virus (HPV), which is mostly acquired by sexual intercourse, is a necessary cause of cervical cancer. Most HPV infections resolve spontaneously, but persistent infection may lead to precancerous lesions, and a delay in treatment of these lesions may progress to cervical cancer. Since it usually takes 10–20 years for precursor lesions to develop into invasive cervical cancer, most cervical cancers can be prevented by early detection and treatment of precancerous lesions [1, 2].

Cervical cancer is the second most common cancer in women worldwide. Greater than 500,000 new cases of cervical cancer and more than 270,000 women die from cervical cancer, which contains 9% of female cancer deaths, every year [3]. In Sub-Saharan Africa, invasive cervical cancer incidence is one of the highest in the world with an age-standardized incidence rate of 31.0 per 100,000 women. Similarly, many women in East, Central and Southern African countries did not use screening services [4, 5].

Studies conducted in Pakistan and Cameron on awareness and knowledge about cervical cancer among health care providers revealed that 61% and 60% respondents responded HPV virus as a cause of cervical cancer [6, 7]. In a study done in Thailand, 36% of nurses and 55.5% of doctors were aware the HPV subtypes [8]. Concerning mode of transmission of HPV infection, the study conducted in Pakistan, 60.6% replied by sexual intercourse and 27.7% by direct genital contact, and in Tanzania 89% replied by sexual intercourse and 10% by blood contact [6, 9].

It is believed that cervical cancer ranks as the most frequent cancer among women in Ethiopia [10, 11]. It was also estimated that every year 4648 and 3235 women diagnosed and die with cervical cancer respectively [12]. A community based cross-sectional study done in North West Ethiopia had showed that 78.7% of respondents had heard about cervical cancer and a small proportion (31%) had good knowledge about the disease [13]. Another facility based study done on reproductive health clients in Addis Ababa had showed that 81.2% had never heard of Pap smear screening and minimal proportion of them (6.5%) had ever had a Pap smear test [14].

There is lack of data on assessment of cervical cancer services and cervical cancer related knowledge of health service providers in Ethiopia. Therefore, this study aimed to assess cervical cancer services and cervical cancer related knowledge of health service providers, and association between socioeconomic status characteristics and knowledge in public health facilities in Addis Ababa.

## Main text

### Study setting and method

The study was conducted in Addis Ababa, which is the capital city of Ethiopia. Administratively, the city is divided into ten sub-cities. There are 48 hospitals and more than 35 health centers. The study implemented facility based cross sectional study design.

### Data collection and analysis procedures

A pretested self-administered questionnaire and a check list were used to assess knowledge of health service providers related to cervical cancer and availability of cervical cancer service facilities. Both instruments were developed by reviewing available relevant literature and WHO guidelines. Three BSc nurses who had previous data collection experience and one BSc nurse who had previous supervision experience were used as data collectors and supervisor respectively. Two days training was given about the purpose of the study, data collection tools, contents and ethical procedures.

Data were coded and entered using Epi Info version 3.5.3 and analysis were performed using SPSS version 21. Frequencies, means and Chi-square test were used to look for an association between cervical cancer knowledge and socio-demographic variables. The overall knowledge of the respondents was assessed using the mean score of the outcome as a cut-off value (21.3). For each question, a score of one was given to correct answer and score of zero was given to the incorrect and I do not know answers. The overall knowledge score was obtained by summing the responses. The composite score was dichotomized using mean obtained from the data. Individuals who scored ≥ 21.3 knowledge score about cervical cancer were classified as having good level of knowledge and those who scored < 21.3 as having poor level of knowledge.

## Results

### Socio-demographic characteristics of the respondents

Of the 343 health care professionals who received the questionnaires, 309 had completed and returned, giving a response rate of 90%. The mean age of the respondents was 29.2 (Standard Deviation ± 7.2) years. Most of the respondents were female 207 (67%). The majority of respondents 191 (61.8%) were single in marital status and 222 (71.8%) were orthodox religion followers. Diploma nurses 80 (25.9%) and degree nurses 99 (32%) accounted majority of respondents. The mean duration of working experience of the respondents was 6.2 (Standard Deviation ± 6.7) years (Table 1).

**Table 1:** Socio-demographic characteristics of the respondents, Addis Ababa, 2014

Characteristics	Frequency	Percent
Age groups (years)		
20–29	210	67.9
30–39	66	21.4
40–49	21	6.8
50–59	12	3.9
Sex		
Female	207	67
Male	102	33
Marital status		
Single	186	60.2
Married	123	39.8
Religion		
Orthodox	222	71.8
Muslim	28	9.1
Protestant	59	19.1
Professional level		
Obstetrician and gynaecologist	6	1.9
Internist	3	1
General practitioner	45	21
Health officer	34	11
Diploma nurse	80	25.9
Degree nurse	99	32
Diploma midwifery	37	12
Degree midwifery	34	11
Work experience (years)		
1–9	255	82.5
10–19	32	10.4
20–29	13	4.2
30–39	9	2.9

### Knowledge of health service providers about cervical cancer risks

Having multiple sexual partners 257 (83.2%) and early sexual debut 168 (54.4%) were the most commonly mentioned risk factors for cervical cancer. About 4% of the respondents reported that they did not know the risk factors for cervical cancer. Besides, the most commonly reported clinical manifestations for cervical cancer were bleeding after intercourse 251 (81.2%) and offensive or blood-stained vaginal discharge 215 (69.6%). On the other hand, nearly 3% of the respondents are not aware of the clinical manifestations of cervical cancer (Table 2).

**Table 2:** Frequency distribution of knowledge of respondents’ cervical cancer risk factors and clinical manifestations, Addis Ababa, 2014

Knowledge of respondents	Frequency	Percent
Risk factors		
Smoking	158	51.1
Multiple sexual partners	257	83.2
Multi parity	100	32.4
Early sexual debut	168	54.4
Long term use of contraceptive pill	117	37.9
Suppressed immunity	141	45.6
I do not know	12	3.9
Clinical manifestations		
Bleeding after intercourse/douching	251	81.2
Inter-menstrual bleeding	126	40.8
Painful coitus	172	55.7
Vaginal discharge	215	69.6
Unexplained weight loss	125	40.5
Anaemia	157	50.8
Swelling of cervix	128	41.4
I do not know	8	2.6

### Knowledge of health service providers about cervical cancer services

Cervical cancer screening, early diagnosis and treatment are the secondary and tertiary prevention options. Pap smear 232 (75.1%), biopsy 262 (84.8%), cryotherapy 108 (35%) and chemotherapy 205 (66.3) were the most frequently answered screening, diagnosis, precancerous and cancer treatment respectively. Majority of the respondents, 291 (70.5%) were aware of the correct time to start screening (women younger than 30 years plus Human Immune-deficiency Virus infected and women between 30 and 49 years), but only 95 (25.9%) knew the correct screening intervals which is every 5 years and every 10 years if HPV test done.

### Association of cervical cancer knowledge and socio-demographic variables

Among the socio demographic variables, profession and sex had a statistically significant association with knowledge about cervical cancer. General practitioner and above (X^2^ = 20.93, P = 0.000) and health officers and degree nurses (X^2^ = 5.90, P = 0.015) had higher knowledge about cervical cancer than diploma nurses. Females had better knowledge about cervical cancer than males (X^2^ = 8.4, P = 0.003) (Table 3).

**Table 3:** Association of cervical cancer knowledge and socio-demographic characteristics, Addis Ababa, 2014

Variables	Good knowledge	Poor knowledge	X^2^	P-value
Profession				
General practitioner and above	21	3	20.93	0.000
Degree nurse	83	85	5.9	0.015
Diploma nurse	40	77		
Work experience (years)				
1–9	120	135		
10–19	15	17	0.03	0.866
20–29	6	7	0.05	0.825
30–39	3	6	0.22	0.637
Age group (years)				
20–29	91	119		
30–39	36	30	2.11	0.146
40–49	14	7	3.30	0.069
50–59	3	9	0.90	0.342
Marital status				
Married	57	66	0.00	0.966
Single	87	99		
Sex				
Female	84	123	8.4	0.003
Male	60	42		

## Discussion

All the hospitals had trained health providers and screening, diagnosis and treatment infrastructures for the available cervical cancer services. Less than half of the respondents knew cervical cancer could be prevented by vaccination and majority of them did not know the recommended age group for HPV vaccine and the recommended number of doses.

The ratio of trained health professional especially general practitioner and above to population is still a problem in Ethiopia, (1:> 50,000) [15]. In this study though, there were trained health professionals for the available cervical cancer services, they engaged with plenty of other health services. Because of this the patients may be deprived from getting timely services and wasting extra resources. A similar study in East, Central and Southern African Countries shared this finding [4].

The majority of the respondents in this study, 62.1% said virus as biological cause for cervical cancer infection and 58.9% mentioned HPV as the name of the virus. Similar results observed in Pakistan 62% said virus and 61% said HPV the name of the virus and in Cameron 60% said HPV the name of the virus [6, 7]. Regarding mode of transmission, 88.7% correctly answered sexual intercourse as one means. In this study majority of the respondents, (93.5%) said cervical cancer infection was a preventable disease and of these, 59.9% said by sexual abstinence, 38.9% said by vaccination and 44.3% said by condom. Other similar studies in Cameron 44% by vaccination, in Tanzania 48% by condom, 22% by vaccination, and in Nigeria 33.1% by condom [6, 9, 16]. Of the respondents who consider cervical cancer infection could be prevented by vaccination, only 39.2% identified correctly the recommended age for HPV vaccine and 72.8% did not know the recommended number of doses and over all time taken. This low result about the vaccine in this and other studies was not surprising in countries where this vaccine is not included in the routine immunization program.

A large proportion of the respondents in this study were able to mention having multiple sexual partners and early sexual debut as risk factors as also indicated in studies in Cameron, Ghana, India and Tanzania [7, 9, 16, 17]. The most widely mentioned clinical manifestations were post-coital bleeding (81.2%) and offensive or blood stained vaginal discharge (69.6%). Results from studies in Ghana, India, Pakistan and Tanzania also support this finding [6, 9, 16, 17]. The most widely identified screening methods were pap smear (75.1%) and Visual Inspection with Acetic acid (VIA) (50.5%). Majority of the respondents were aware of the correct time to start screening (70.5%), but only small percent (25.9%) knew screening intervals. Similarly, studies in Pakistan, Uganda, Cameron, Ghana, and Tanzania [6, 7, 9, 17, 18]. It was critical that the majority of the respondents were not aware about VIA although it is less costly and simple to perform. Although, precancerous treatment is the best options especially for resource limited countries where there is no good cancer treatment, only 35% mentioned cryotherapy and 27.5% Loop Electrosurgical Excision Procedure (LEEP) as, precancerous treatment options. There was also a significant association between professional background and knowledge of cervical cancer. General practitioners and above were more knowledgeable than nurses as also reported in studies in Thailand [8].

Majority of the respondents knew the biological cause, mode of transmission, risk factors, clinical presentations, screening methods and time to start and prevention methods of cervical cancer. But most of the respondents did not know HPV vaccine, the recommended age group and the recommended number of doses and over all time taken, screening intervals and the precancerous treatment methods. Since health providers play vital role in the prevention of cervical cancer through educating individuals, appropriate training programs for health providers should be designed and provided.

## Limitations

The limitations of the study were that it only included health professionals view and it was mainly descriptive it failed to detect the significant factors associated with the problem. Social desirability bias and recall biases may be also introduced.

## Data Availability

The datasets used and/or analyzed during the current study are available from the corresponding author on reasonable request.

## Abbreviations

HPV: human papilloma virus
VIA: visual inspection with acetic acid
LEEP: loop electrosurgical excision procedure

## References

[1] World Health Organization (2006). Comprehensive cervical cancer control: a guide to essential practice.

[2] World Health Organization (2005). Report of the consultation on human papilloma virus vaccines geneva.

[3] World Health Organization (2013). Comprehensive cervical cancer prevention and control: a healthier future for girls and women.

[4] Zvavahera MC, Simbarashe R (2001). Situation analysis for cervical cancer diagnosis and treatment in East, Central and Southern African countries. Bull World Health Organ.

[5] Karly S, Louie L (2009). Epidemiology and prevention of human Papilloma virus and cervical cancer in sub-Saharan Africa: a comprehensive review. Trop Med Int Health.

[6] Ali S, Ayub S (2010). Knowledge and awareness about cervical cancer and its prevention amongst interns and nursing staff in Tertiary Care Hospitals in Karachi, Pakistan. PLoS ONE.

[7] McCarey C, Pirek D (2011). Awareness of HPV and cervical cancer prevention among Cameroonian healthcare workers. BMC Women’s Health.

[8] Songthap A, Pitisuttithum P (2009). Knowledge, attitudes, and acceptability of a human papillomavirus vaccine among healthcare providers, Bangkok, Thailand. Southeast Asian J Trop Med Public health.

[9] Urasa M, Darj E (2011). Knowledge of cervical cancer and screening practices of nurses at a regional hospital in Tanzania. Afr Health Sci.

[10] Fantahun M, Berhane Y, Tsui A. Text book of reproductive and child health with focus on Ethiopia and other developing countries.

[11] Federal Ministry of Health. National reproductive health strategy, Addis Ababa, Ethiopia, 2006–2015.

[12] World Health Organization/ICO information center. Human papilloma virus and related cancers in Ethiopia. Fact sheet, 2010.

[13] Getahun F, Mazengia F (2013). Comprehensive knowledge about cervical cancer is low among women in Northwest Ethiopia. BMC Cancer.

[14] Terefe Y, Gaym A. Knowledge, attitude and practice of screening for carcinoma of the cervix among reproductive health clients at three teaching hospitals, Addis Ababa, Ethiopia. Ethiopian J Reprod Health. 2008;2(1).

[15] Federal Ministry of Health. Health and health related indicators. Addis Ababa; 2014.

[16] Shah V, Vyas S, Singh A, Shrivastava M. Awareness and knowledge of cervical cancer and its prevention among the nursing staff of a tertiary health institute in Ahmedabad, Gujarat, India. ecancermedicalscience. 2012;6. 10.3332/ecancer.2012.270 PMC3437739 23008746

[17] Adageba R, Danso K (2011). Knowledge of cervical cancer and patronage of cervical cancer screening services among female health workers in Kumasi, Ghana. Afr J Haematol Oncol.

[18] Mutyaba T, Mmiro FA, Weiderpass E (2006). Knowledge, attitudes and practices on cervical cancer screening among the medical workers of Mulago Hospital, Uganda. BMC Med Educ.

